# Comparative Proteomic Analysis of Ridge Gourd Seed (*Luffa acutangula* (L.) Roxb.) during Artificial Aging

**DOI:** 10.1021/acsomega.4c01270

**Published:** 2024-05-30

**Authors:** Jakkaphan Kumsab, Yodying Yingchutrakul, Nattapon Simanon, Chonchawan Jankam, Chutima Sonthirod, Sithichoke Tangphatsornruang, Chutikarn Butkinaree

**Affiliations:** National Center for Genetic Engineering and Biotechnology, National Science and Technology Development Agency, Pathum Thani 12120, Thailand

## Abstract

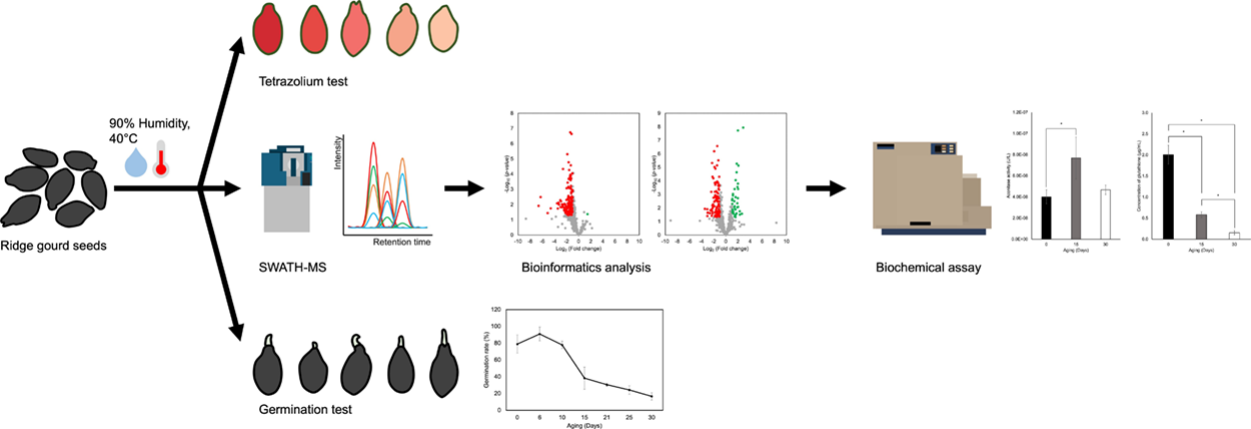

Seed aging is a complicated process influenced by environmental conditions, impacting biochemical processes in seeds and causing deterioration that results in reduced viability and vigor. In this study, we investigated the seed aging process of ridge gourd, which is one of the most exported commercial seeds in Thailand using sequential window acquisition of all theoretical fragment ion spectra mass spectrometry. A total of 855 proteins were identified among the two groups (0 d/15 d and 0 d/30 d). The Gene Ontology and Kyoto Encyclopedia of Genes and Genomes analyses of differentially expressed proteins revealed that in ridge gourd seeds, the aging process altered the abundance of proteins related to the oxidative stress response, nutrient reservoir, and metabolism pathway. The most identified DEPs were mitochondrial proteins, ubiquitin–proteasome system proteins, ribosomal proteins, carbohydrate metabolism-related proteins, and stress response-related proteins. This study also presented the involvement of aconitase and glutathione pathway-associated enzymes in seed aging, with aconitase and total glutathione being determined as possible suggestive biomarkers for aged ridge gourd seeds. This acquired knowledge has the potential to considerably improve growing methods and seed preservation techniques, enhancing seed storage and maintenance.

## Introduction

*Luffa acutangula* (L.) Roxb., usually known as ridge gourd, angled luffa, or ribbed gourd,^1^ is a medicinal plant and food that is widely grown in Southeast Asia, India, China, Japan, Egypt, and other African countries.^2^ Ridge gourd belongs to the family Cucurbitaceae, subfamily Cucurbitoideae, Tribe Benincaseae, Subtribe Luffinae, and genus *Luffa*. It is renowned for its high nutritional value due to its abundance and diversity of nutrients and is rich in vitamin C, riboflavin, niacin, and essential amino acids.^3^ This plant is widely used in the traditional medicinal system to treat various health conditions including jaundice, diabetes, hemorrhoids, dysentery, headache, ringworm infection, and leprosy.^4^ Ridge gourd is one of the cucurbit crops that contribute significantly to world food and nutrition security and is economically essential to smallholder farmers, who account for 83% of global cucurbit production.^5^ Thailand has been the leading exporter of ridge gourds among all Asian countries, with a particularly strong position in the competitive Western European market.^6,7^ Moreover, the demand for ridge gourds in Asia is continuously increasing.^8,9^ The cultivation demand is increasing due to the nutritional requirements of a growing population.^10^ To produce high-quality commercial ridge gourd fruit and seed in order to meet worldwide demand, a comprehensive study of the biological processes (BPs) of the ridge gourd seed is essential to enhance production efficiency and improve seed quality.

Seed aging is a process that causes the quality of seeds to deteriorate, resulting in reduced germination and vigor. This process can occur during seed development, after harvesting, and during storage, and it can be influenced by various environmental factors, such as temperature, humidity, and oxygen availability.^11−13^ Aged seeds have been reported to cause a decrease in antioxidant systems,^14^ disruption of cellular membranes,^15^ genetic integrity damage,^16^ lipid peroxidation,^17^ and protein degradation in seeds.^18^ Elevated temperatures have been identified as a significant factor that expedites the aging process of seeds, subsequently diminishing their viability.^19,20^ The effect of temperature stress on the seed germination rate and vigor has been reported. The study on sunflower seeds reported that high temperatures can cause seed stress and enzyme activity dysregulation. It may reduce seed vigor by suppressing storage substance catabolism in the seed endosperm.^21^ The report of the maize seed artificial aging study revealed that the germination energy, germination rate, and stress-related enzyme activities were reduced during artificial aging.^22^ The previous study on soybean seeds reported that artificial aging is correlated with mitochondrial activities and the antioxidant system.^23^ Several studies have been conducted to investigate the effects of seed aging on various aspects of seed physiology, biochemistry, and molecular biology, using a range of experimental approaches and techniques.^24−28^ To increase seed quality, extend the expiration date and ensure agricultural yield and food security, it is essential to understand the mechanisms driving seed aging.^29^

Recently, proteomic analysis has emerged as a powerful tool for investigating the changes in protein composition and abundance during seed aging.^30^ Proteomic studies of seed aging have led to a deeper understanding of the underlying mechanisms and identified potential targets for improving seed storage and preservation. The proteomic study has the potential to provide novel insights into the process of seed storability, which can facilitate a comprehensive understanding of seed longevity.^31^ Even though proteomic techniques like two-dimensional electrophoresis (2-DE) have been widely employed to investigate seed physiology, these gel-based approaches are restricted in their capacity to detect low abundance and hydrophobic proteins, as well as their accuracy and repeatability.^32,33^ These limitations can be overcome by utilizing SWATH-MS as a quantification tool in gel-free mass spectrometry-based quantitative proteomic approaches.^34,35^ Hence, SWATH-MS has come to be the technology of choice for high-throughput protein and proteome characterization.^36^

In this study, we conducted artificial aging and germination tests to investigate the physiochemical response of the ridge gourd seeds. Biochemical changes in artificially aged ridge gourd seeds were observed by using tetrazolium (TZ) seed staining. SWATH-MS quantitative proteomic analysis was acquired to characterize the ridge gourd seeds during artificial aging. We performed bioinformatic analysis to categorize the differentially expressed proteins (DEPs) into processes and pathways. The biochemical activity assay was employed to confirm the DEPs. The identified DEPs were associated with diverse functions including mitochondrial proteins, ribosomal proteins, carbohydrate-related proteins, and stress response-related proteins. The results from this research contribute to improving the comprehension of seed deterioration and the identification of potential biomarkers that prevent seed deterioration. Therefore, this study can be used to develop and evaluate techniques for improving the quality of the ridge gourd seeds.

## Results

### Effect of Artificial Aging on the Germination of Ridge Gourd Seeds

**Figure 1:**
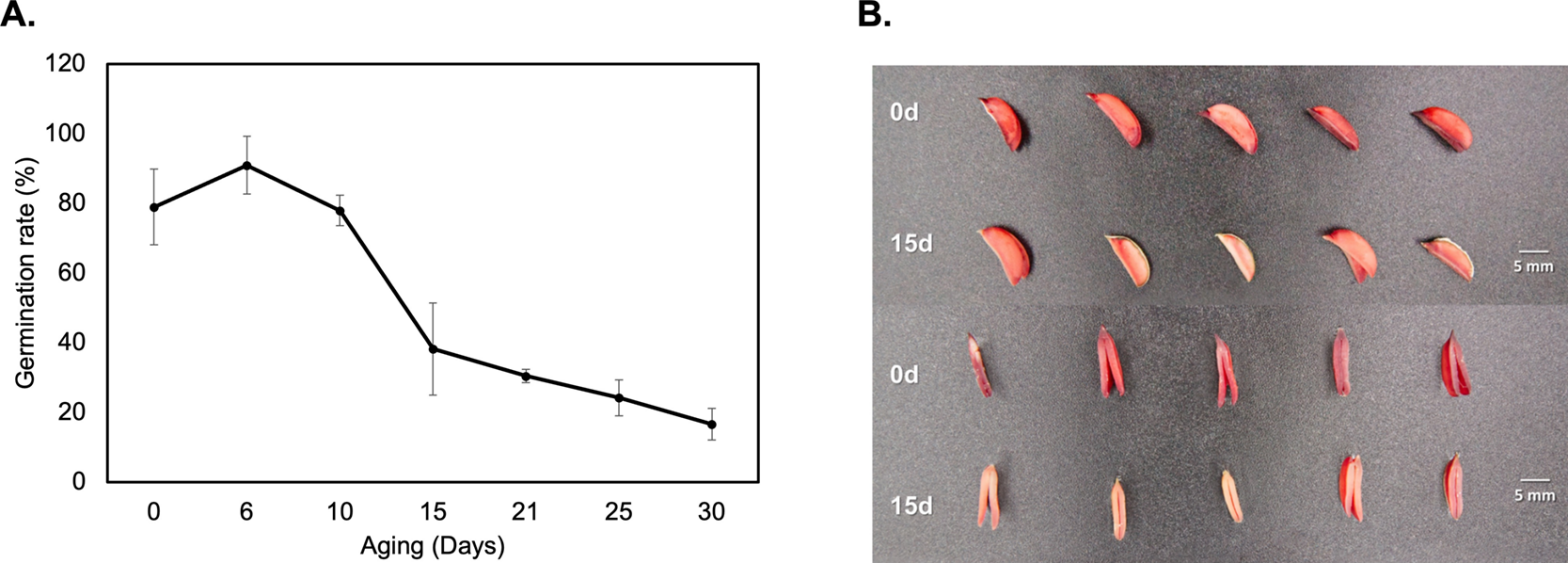
Seed germination test and TZ test. (A) Effect of artificial aging treatment on the ridge gourd seed germination rate. Values represent the means ± SD from three biological replications. (B) Effect of artificial aging treatment on metabolism of ridge gourd seeds nonage seed (0 d) vs artificial aged seed (15 days).

The germination rate was investigated to identify physiological changes that occurred from artificial aging (Figure 1A). The germination rate of seeds following artificial aging treatment slightly increased from 79 to 91% after 6 days of aging. However, the germination rate declined to 78% at 10 days, 38% at 15 days, 31% at 21 days, and 24% at 25 days, eventually reaching 16% at 30 days. According to our seed germination test results, we chose to perform proteomic and biochemical analyses on seeds that have been aged for 15 and 30 days as their germination rate dropped by more than 50%. Ridge gourd seeds aged 15 days were chosen for proteomic and biochemical tests based on their germination rate. The ridge gourd seeds from 0 and 15 days were examined using the TZ test to assess the difference in seed respiration activities. As shown in Figure 1B, 15-day seed metabolism was obviously reduced by the aging process. Herein, the TZ test results were correlated with the germination rate, suggesting that artificial aging decreased the total respiration enzyme activities and the germination rate of ridge gourd seed.

### Proteomic Analysis Reveals a Significant Change in Ridge Gourd Seed Proteome after Artificial Aging

**Figure 2:**
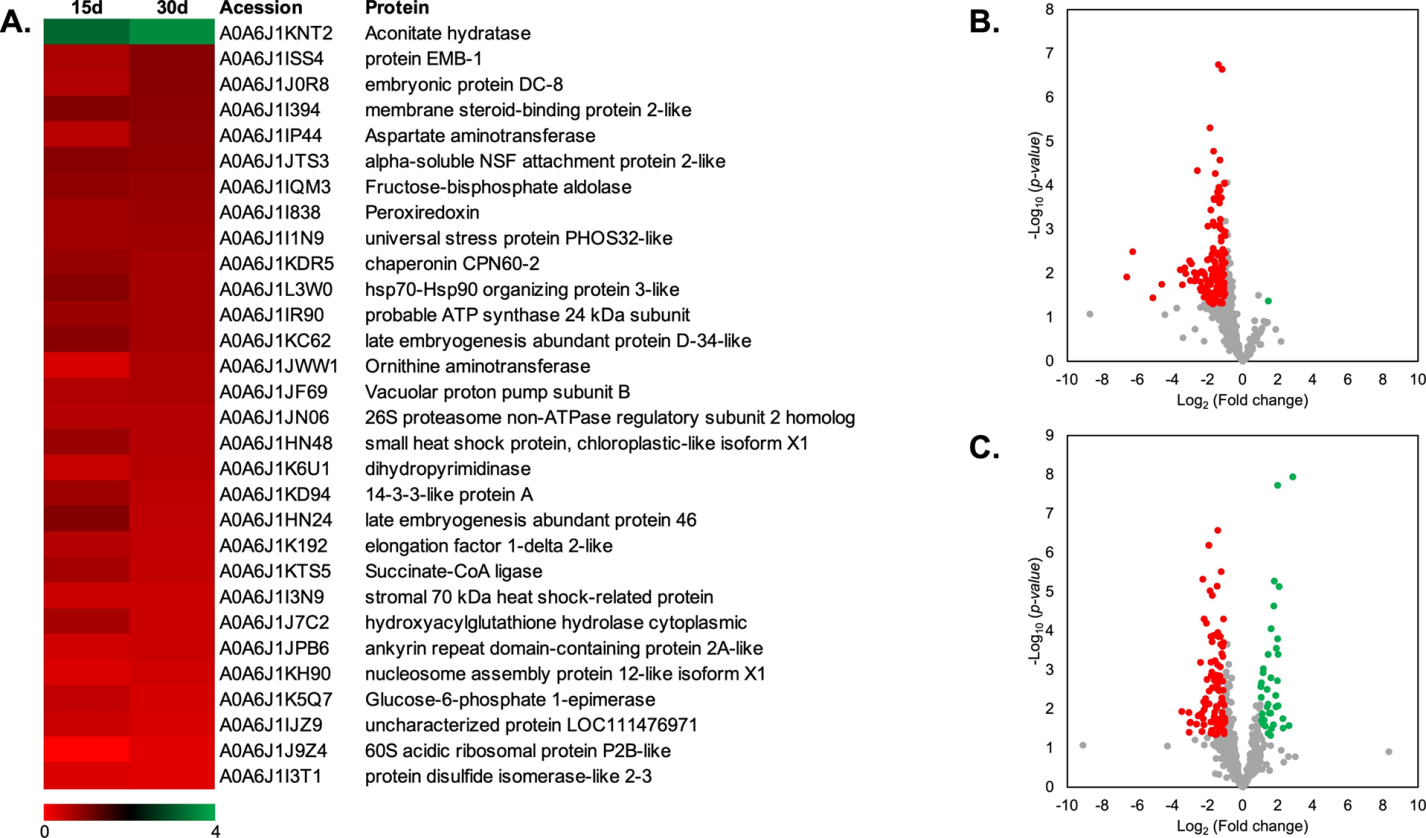
Proteomic analysis of ridge gourd seed. (A) Protein quantification of DEPs heatmap showing DEPs presented on 15 and 30 days. (B, C) Volcano plot shows DEPs in 0 d/15 d and 0 d/30 d. The log_2_ (fold change) denoting the magnitude of FCs (*x* axis) was plotted against −log_10_ (*p*-value), representing the statistical significance (*y* axis). Red and green dots represent significant down and up expression proteins, respectively. Gray dot represents nonsignificant data. Data were derived from three biological replications and three technical replications for each biological replication.

To better understand how ridge gourd seed proteomes changed throughout the aging process, three groups of ridge gourd seed samples were prepared: nonaged seed (0 d), 15-day artificial aged seed (15 d), and 30-day artificial aged seed (30 d) to perform SWATH-MS-based proteomic analysis. A total of 3765 proteins were identified against the Cucurbitaceae protein database from the spectral library (Table S1). Using 0 day as a control, two comparison groups (0 d/15 d and 0 d/30 d) were constructed to identify DEPs. Proteins with a fold change (FC) ≥ 2.0 or ≤0.5 with p-value <0.05 were considered significantly upregulated and downregulated, respectively. In this study, 6 and 213 proteins were upregulated in 15 and 30 days, respectively (Table S2), while 491 and 366 proteins were downregulated in 15 and 30 days, respectively (Table S3). To emphasize the dynamic changes of proteins occurring during aging processes, heatmap reveals DEPs of FC of protein expression in 15 and 30 days versus 0 day as a control. Our results demonstrated that aconitase (Aconitase hydrates: A0A6J1KNT2) was the only protein that progressively increased after 15 and 30 days. Other proteins were found to significantly decrease on 15 and 30 days (Figure 2A). Furthermore, the volcano plot was utilized to show DEPs in 15 d/0 d and 30 d/0 d. As shown on the volcano plot, we observe that most of the DEPs were downregulated on both 15 and 30 days/0 days (Figure 2B,C).

### GO and KEGG Analysis of Proteomics Changed in Artificial Aged Ridge Gourd Seed of 15 and 30 Days

**Figure 3:**
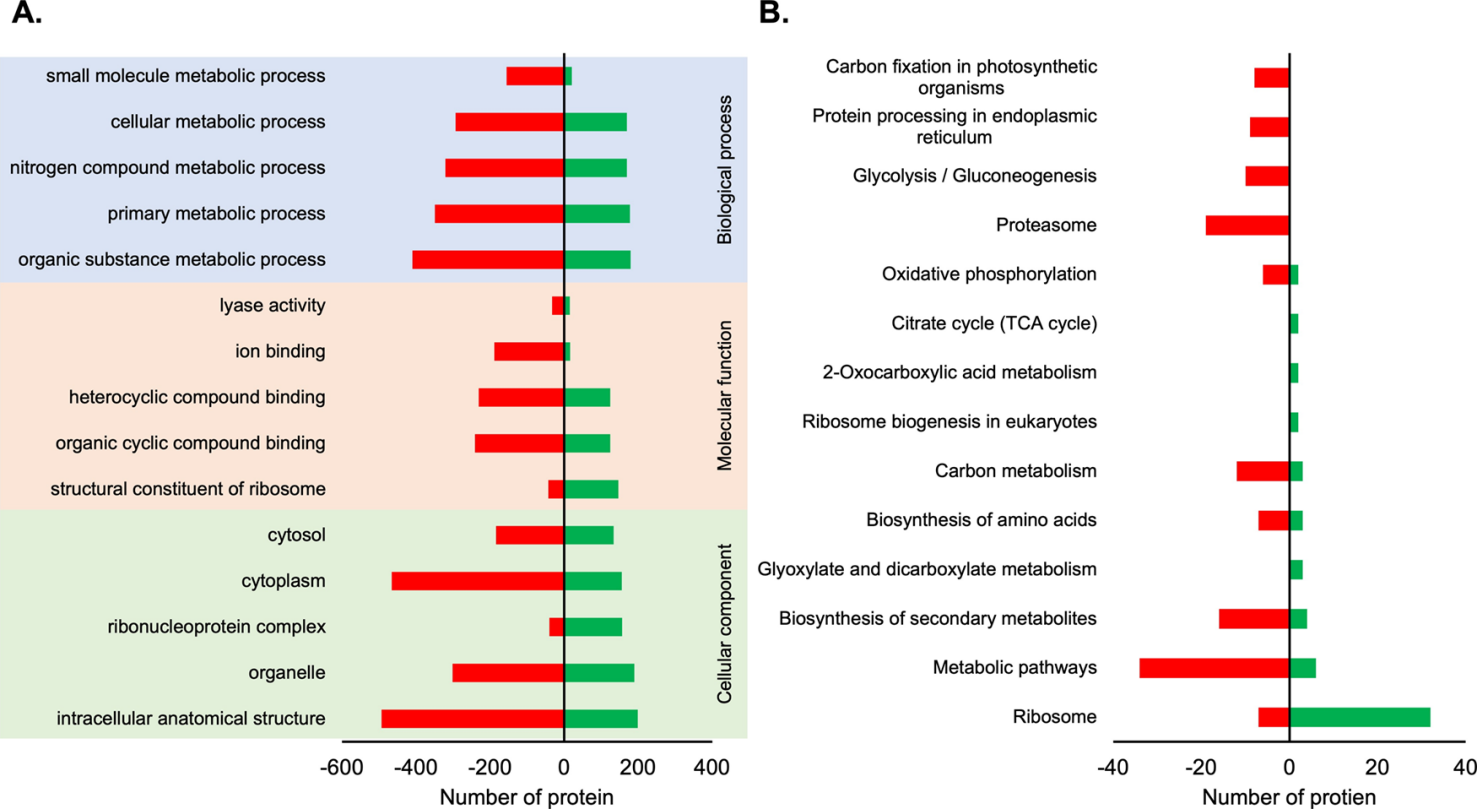
GO classification and KEGG analysis of ridge gourd seed proteome. (A) GO classification of ridge gourd up- and downregulated DEPs of 15 and 30 days, indicating proteins in BP, MF, and CC. (B) KEGG analysis of ridge gourd seed presented the up- and downregulated DEPs. Red and green bar graphs represent significant down- and up-expression protein, respectively.

Gene Ontology (GO) annotation and Kyoto Encyclopedia of Genes and Genomes (KEGG) analyses were performed.^37−39^ The objective of GO analysis was to conduct enrichment analysis on gene sets and classified into three major categories: BP (Figure S1), molecular function (MF) (Figure S2), and cellular component (CC) (Figure S3). The proteomic results revealed that upregulated DEPs of BP related to the organic substance metabolic process, primary metabolic process, cellular metabolic process, nitrogen compound metabolic process, and biosynthetic process. In the category of BP, the downregulation of DEPs was mainly involved in the organic substance metabolic process, primary metabolic process, nitrogen compound metabolic process, cellular metabolic process, and small molecule metabolic process (Figure 3A). In terms of MF, the upregulated group was primarily related to structural constituents of the ribosome (Figure S2), heterocyclic compound binding, and organic cyclic compound binding (Figure 3A). Regarding the MF of the downregulation group, DEPs were associated with organic cyclic compound binding, heterocyclic compound binding, ion binding, small molecule binding, and hydrolase activity (Figures 3A and S2). According to CC category, the upregulated DEPs were involved in intracellular anatomical structure, organelle, ribonucleoprotein complex, cytoplasm, and cytosol. The downregulation of DEPs of CC was mainly related to intracellular anatomical structure, cytoplasm, and organelles (Figure 3A).

KEGG analysis was performed using the *Cucurbita maxima* (winter squash) database (the highest similarity database to ridge gourd). Most DEPs were found to be implicated in pathways related to the metabolic system. The KEGG analysis data demonstrated that ribosomal proteins were the predominant upregulated proteins (Table S4). On the other hand, most of the decreased proteins belonged to the metabolic pathway (Figure 3B). Downregulated DEPs were involved in the metabolic system. In addition, we found that the proteasome and secondary metabolic pathway were also downregulated. For upregulated DEPs, proteins were mainly involved in ribosome function (Table S4).

### Protein–Protein Interaction Analysis of DEPs

**Figure 4:**
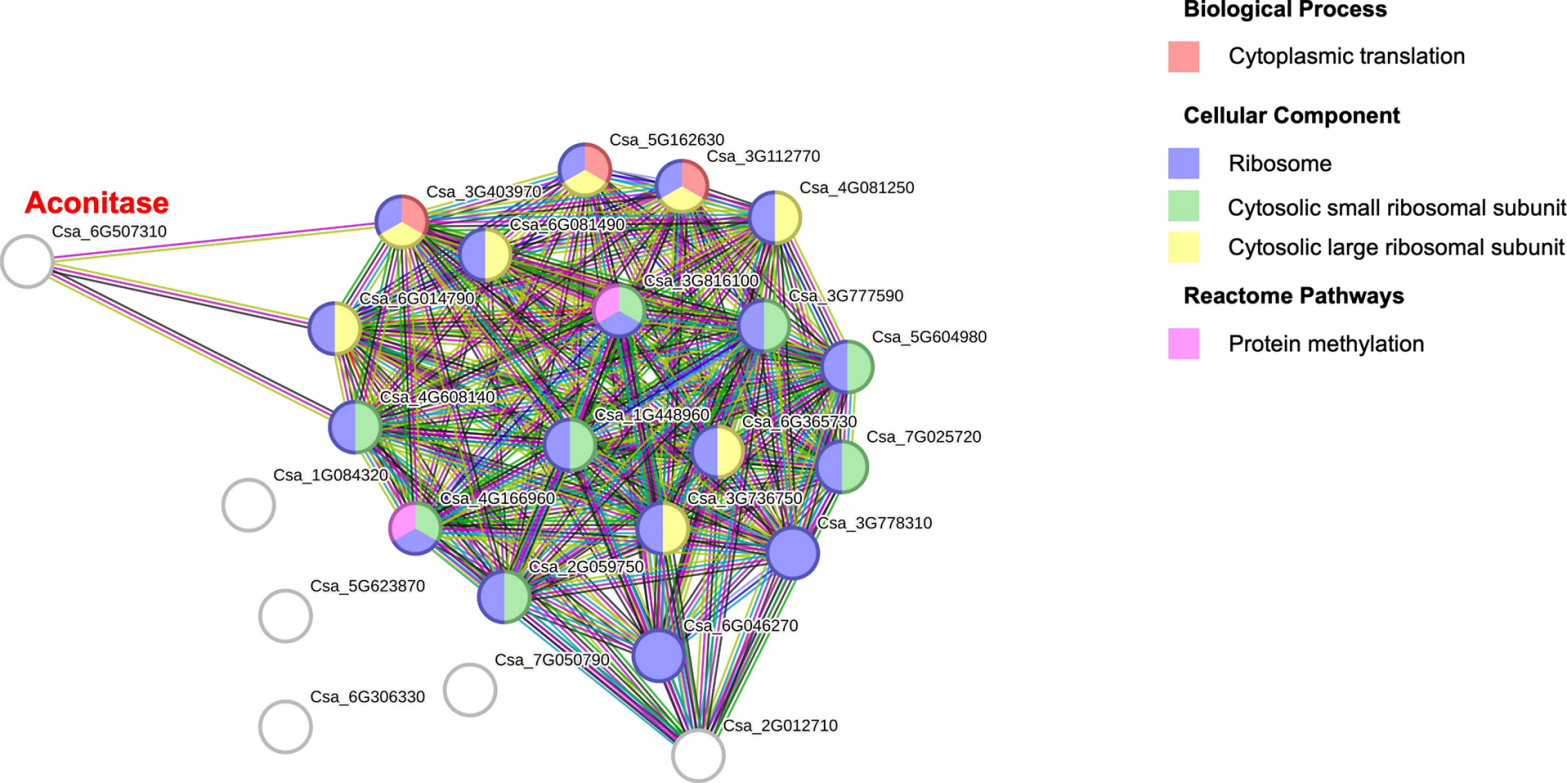
STRING analysis reveals the protein–protein interaction of the upregulated DEPs of 30 days.

To understand the connectivity within a biological system of aged ridge gourd seeds, we performed a protein–protein interaction analysis of DEPs to identify interactions between proteins. The STRING analysis of downregulated DEPs of 15 days revealed that the highly connected subnetworks, including the citrate cycle and glycolysis/gluconeogenesis, were enriched and visualized (Figure S4). In addition, the ribosome system was also enriched with upregulated DEPs for 30 days. Aconitase was found to connect with the ribosomal proteins after 30 days of artificial aging, which interacts with three of the ribosomal proteins (Figure 4). The enrichment of the proteasome network found abundant proteins in downregulated DEPs within 30 days of seed aging analysis (Figure S5).

### Oxidative Stress Regulation Was Involved in the Aging of Ridge Gourd Seeds

**Figure 5:**
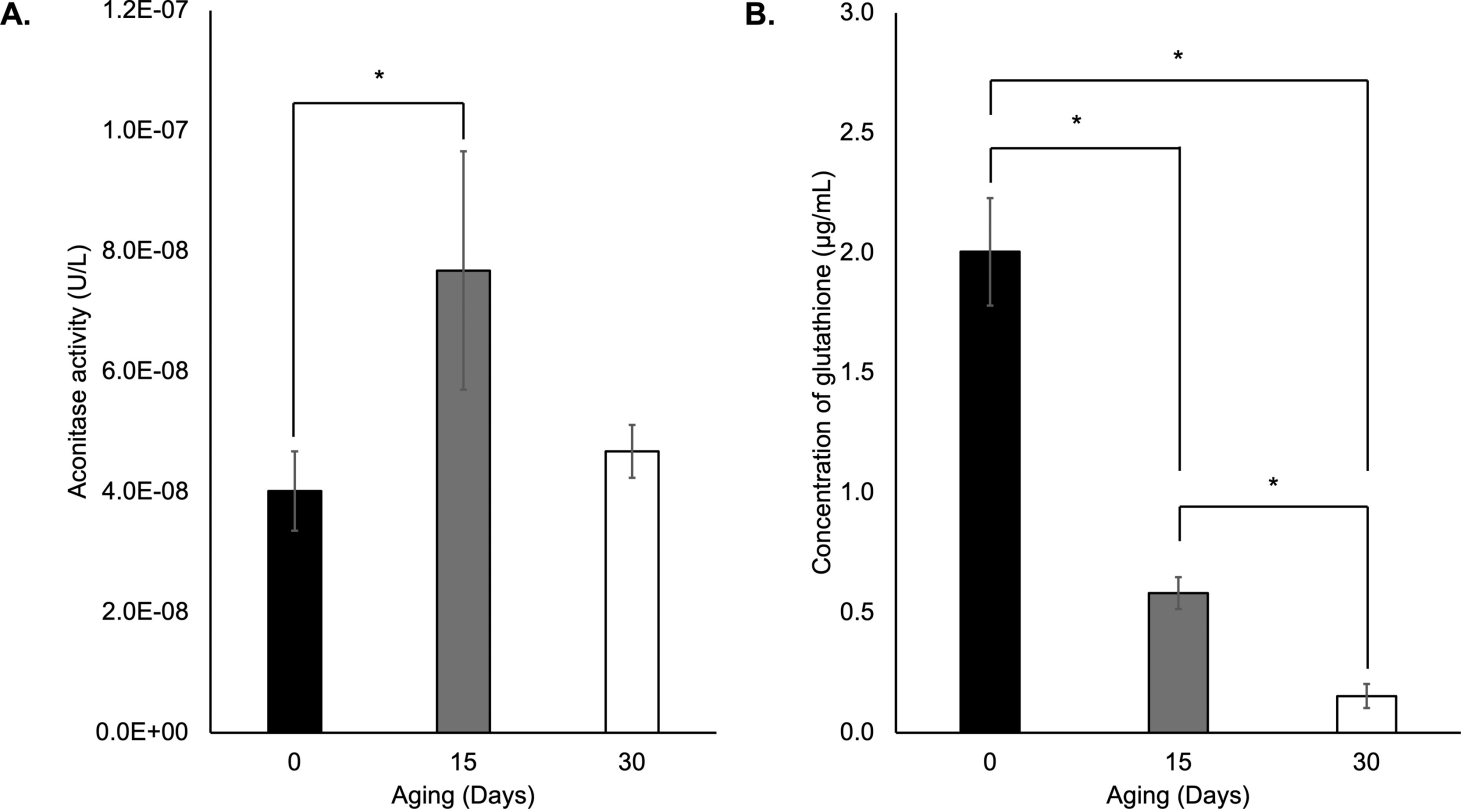
Biochemical test. (A) Aconitase activity. (B) Total glutathione content. The data represent mean ± standard deviation of three biological replications and three technical replications for each biological replication. Black, gray, and white bars represent 0, 15, and 30 days, respectively. Statistically significant differences between control and treatments were analyzed by Student’s *t* test (*p* < 0.05).

According to the SWATH-MS results, we observed that the expression of aconitase and glutathione-related proteins was consistently changed during artificial aging. We therefore investigated the aconitase activity and total glutathione concentration to determine whether they could be employed as indicators for ridge gourd seed aging. To validate the aconitase activity and glutathione content, 0 and 15 days seed samples were selected to study. As shown in Figure 5A, aconitase activity significantly increased by 1.9-fold in 15 days. Moreover, we observed an increase in aconitase activity on 30 days by 1.2-fold compared to 0 day. In addition, total glutathione was significantly decreased by 3.4-fold on 15 d compared to the control group and dramatically decreased until 30 d by 13.1-fold (Figure 5B). This suggests that artificially aged treatment of ridged gourd seed strongly impacts aconitase activity and total glutathione content.

### Determination of the Sugar Content in Ridge Gourd Seeds

**Figure 6:**
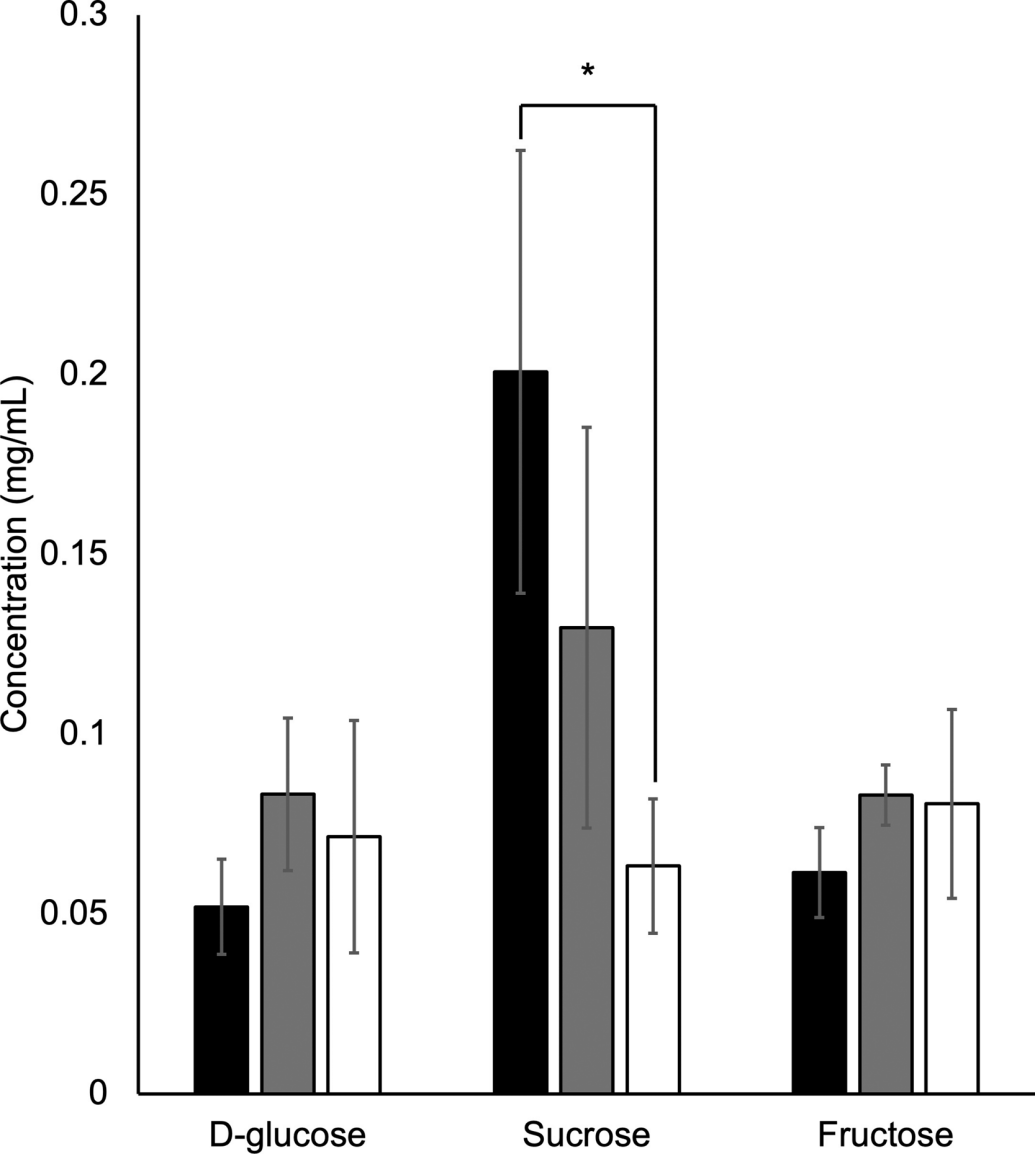
Sugar content. The data represent mean ± standard deviation of three biological replications and three technical replications for each biological replication. Black, gray, and white bars represent 0, 15, and 30 days, respectively. Statistically significant differences between control and treatments were analyzed by Student’s *t* test (*p* < 0.05).

The proteomic results showed that DEPs were associated with the sugar pathway and carbon metabolism. The key enzymes present in these pathways exhibited significant changes in expression levels. Further study of this sugar metabolism is crucial for a comprehensive understanding of its effect on cellular processes. The sugar content was measured to understand the influence of artificial aging on ridge gourd seeds. Our results demonstrated that d-glucose and fructose slightly increased on 15 days of artificially aged seed. We noticed that d-glucose and fructose decreased in 30 days compared to 15 days. On the other hand, sucrose showed a decreasing level, indicating the effect of seed aging. The sucrose content decreased on 15 days and significantly decreased on 30 days (Figure 6).

## Discussion

The present study used comparative proteome analysis to investigate the differential expression of proteins from artificially aged ridge gourd seeds. In addition, bioinformatic studies (KEGG, GO, and STRING) and biochemical assays were employed to analyze the DEPs. In order to comprehend the BPs involved in ridge gourd seed aging processes, the DEPs were classified into five groups based on their recognized activities in protein production and the pathway, including aconitase, ribosomal proteins, carbon metabolism-related proteins, glutathione metabolism-related proteins, and the ubiquitin–proteasome system-related proteins.

To select the appropriate artificial aging time point for the proteomic investigation, we performed a seed germination test to evaluate the germination rate. We observed a rising rate of germination after 6 days of aging. This minor variation in germination rate might be related to heat-induced oxidative stress, implying that increased reactive oxygen species (ROS) may be positive for seed germination and seedling growth,^40^ resulting in a negligible increase in the germination rate. Germination rates decreased gradually after day 6 of artificial aging. Thus, we collected seeds at 0 d (79%), 15 d (38%), and 30 d (16%) to investigate proteomics. According to Thailand’s minimum seed certification criteria regulated by the Department of Agriculture (DOA) and the Ministry of Agriculture and Cooperatives (MOAC),^41^ commercial ridge gourd seeds must have a germination rate of at least 75% to pass the germination standard, a point we selected as it aligns with Thai seed law for selling commercial seed.

In this work, we used TZ testing to determine the difference in seed respiration activities between ridge gourd seeds at 0 and 15 days. From previous studies in barley seeds, TZ testing demonstrated that deteriorated seeds had a lighter color shade than vigor seeds.^42,43^ Similar results were shown in this study, with the stained color of the aged seed being lighter than the nonaged seed (Figure 1B), indicating aging influenced the cellular respiration of ridge gourd seed, as a lower respiratory rate was a characteristic of seed aging and degradation in other seed species, such as soybean^44^ and wheat seeds.^45^

The quantitative proteomic results revealed that among all the proteins detected by SWATH-MS, only aconitase (Aconitate hydratase: A0A6J1KNT2, A0A6J1IM56, A0A6J1GF86, A0A6J1EGD5, A0A1S3B2W8, and A0A0A0KHD6) increased by 2.8-folds on 15 days (15 d) and 3.4-folds on 30 days (30 d). The aconitase expression was dependent on the duration of the artificial aging. This observation indicated that aconitase levels may reflect the stress of high temperature and humidity conditions. Pathway analysis against the KEGG database of DEPs demonstrated that upregulated aconitase was involved in many pathways including metabolic pathways, biosynthesis of secondary metabolites, glyoxylate and dicarboxylate metabolism, biosynthesis of amino acids, carbon metabolism, 2-oxocarboxylic acid metabolism, and citrate cycle (Table S4). Aconitase is highly conserved and present in a wide variety of living organisms^46^ and has been identified in several plant tissues.^47^ The enzyme exists in two isoforms, with the mitochondrial isoform being a component of the tricarboxylic acid (TCA) cycle. The other isoform exists in the cytosol and is involved in the glyoxylate cycle.^48,49^ During the TCA cycle, aconitase catalyzes the reversible isomerization of two TCAs, citrate and isocitrate.^50^ Aconitase is one of the rare proteins that has an iron–sulfur cluster with no electron transport activity. During a Fenton reaction in the presence of iron and sulfur, aconitase in the mitochondrial matrix is able to transform hydrogen peroxide into hydroxyl radicals.^51^ Commonly, aconitase is a catalytic enzyme that belongs to the citrate cycle in plants. Interestingly, this function may mainly involve the stress mechanism of the ridge gourd against oxidative stress during the aging process as the main function. The enzyme activity of the plant aconitase may function as an oxidant biosensor. There have been several studies that have reported the effect of aging on aconitase. The study of stress in *Arabidopsis* cell cultures has shown that oxidative stress causes degradation of aconitase, which significantly decreases respiration performance.^52^ However, there have been reports on the upregulation of aconitase. In the proteome studies of aged coix seeds, aconitase was shown to be upregulated at 5 months and downregulated at 10 months of aged seeds.^53^ In addition, the level of mitochondrial aconitase activity in *Crassostrea virginica*, a marine ectotherm, increased as the temperature increased. The development of ROS and oxidative stress were produced in stress *Crassostrea virginica*. This indicates that mitochondrial aconitase is a sensitive indicator of oxidative stress.^54^ In this study, we measured aconitase activity to compare 0 and 15 days (Figure 4). We found that the aconitase activities were correlated with our proteomic study. However, the decrease in aconitase activity in 30 days might be attributed to enzyme denaturation caused by the artificial aging environment. These results suggest that increased aconitase levels correlate with increased resistance to oxidative stress. Protein expression levels of aconitase may be related to the level of oxidative stress, which played a significant role in detoxifying oxidative stress in ridge gourd seed.

According to the KEGG study, most upregulated DEPs were involved in the ribosomal protein-related pathway in aged ridge gourd seed. Many reports showed that ribosomal proteins were often downregulated in aged seeds. Ribosomal proteins have previously been reported to play a crucial role in seed aging processes, as 40S and 60S ribosomes are essential apparatus for protein synthesis.^55^ In addition, aging causes a decrease in the levels of expression of 40S and 60S ribosomal proteins. Hence, the expression levels of most ribosomal subunits were decreased during the aging.^56^ In wheat and oat seeds, accelerated aging resulted in decreased abundance of chromatin and ribosomal proteins, including 40S and 60S ribosomal proteins.^57^ Previous study reported that oxidative stress was increased by artificial aging in pea seed and was suggested as an effector that slowed down or decreased the synthesis of ribosomal proteins, especially 40S and 60S, in order to allow time for repair of nucleic acid damage prior to translation.^58^ A similar trend was also observed in *Arabidopsis* proteome and was suggested to delay protein synthesis and germination to allow seed to nucleic acid damage.^30^ In this work, the proteomic study showed the relevance of 40S and 60S during the aging process of the ridge gourd seed. We discovered that 60S and 40S ribosomes were more likely to be upregulated (32 DEPs) than downregulated (7 DEPs) (Table S4). Ribosomal proteins of 15d were mostly downregulated and subsequently upregulated on 30d. In this research, KEGG analysis results indicated that seed deterioration was related to the protein synthesis system. The effect of artificial aging conditions led to a decrease in the level of ribosomal protein on day 15. Interestingly, our proteomic data on ribosomal protein expression differed from those of prior reports.^56,58^ The artificially aged ridge gourd seed expressed mainly ribosomal protein for 30 days, suggesting the ridge gourd seed may produce ribosomal protein to maintain the protein synthesis system during stress conditions.

Our proteomic studies showed that ridge gourd seed aging was correlated with sugar and carbon metabolism, as we observed in aged ridge gourd seeds decreased expression of enzymes involved in glycolysis, one of the key sources of energy for germination.^59^ In this study, we found that fructose-bisphosphate aldolase protein (A0A6J1I9D2, A0A6J1IQM3, A0A6J1J4H1, and A0A6J1L1K8) continuously decreased during aging treatment, suggesting fructose-bisphosphate aldolase, which catalyzes the conversion of fructose 1–6-diphosphate to glyceraldehyde 3-phosphate and dihydroxy-acetone phosphate, as a target of the seed aging process.^60^ Under the stress of a seed aging environment, the energy supply chain might be affected by the downregulation of these essential proteins in the starch and sucrose metabolic pathways.^61^ According to KEGG analysis, fructose-bisphosphate aldolase was involved in several pathways including metabolic pathways, biosynthesis of secondary metabolites, carbon metabolism, glycolysis, carbon fixation, pentose phosphate pathway, fructose, and mannose metabolism (Table S4). To determine the effect of artificial aging on the sugar content of ridge gourd seed, sugar assays were performed. The result revealed that the fructose and glucose contents increased during artificial seed aging. On the other hand, sucrose content decreased under an aging environment. Seeds rely on nutrients received from the mother plant in the form of carbohydrates, typically sucrose. When sucrose is cleaved, it produces hexose phosphate, which can enter the glycolytic or pentose phosphate pathways.^62^ Soluble sugars have been reported to decrease with seed age.^63^ According to research on accelerated aging sunflower seeds, the soluble sugar, fructose, and sucrose levels in sunflower seeds were dramatically reduced after 12 months of storage.^21^ Moreover, the study of the variations in sugar concentration in aging safflower seeds discovered that sucrose content decreased slowly with age whereas raffinose and monosaccharides content reduced rapidly at 10 days.^64^ This observation of sucrose reduction was consistent with the results of our study. In this research, our study indicated that a deficient sugar- and carbon metabolism-related enzyme may decrease cell energy and result in seed vigor loss during seed deterioration.

Our study showed that the abundance of proteins involved in glutathione metabolism of artificial aging ridge gourd seeds. Artificial aging showed an impact on glutathione metabolism at 15 and 30 days of seed aging, according to proteomic analysis. To gain a better understanding of this phenomenon during seed aging, we performed pathway enrichment analysis via KEGG on the DEPs. Glutathione-related DEPs were found to be a downregulated DEPs group involved in the metabolic pathway (probable phospholipid hydroperoxide glutathione peroxidase (A0A6J1I4M6), probable glutathione S-transferase (A0A6J1IX51), and probable phospholipid hydroperoxide glutathione peroxidase (A0A6J1K6F2)) and glutathione metabolism (probable phospholipid hydroperoxide glutathione peroxidase (A0A6J1I4M6), 1-Cys peroxiredoxin A isoform X1 (A0A6J1I838), probable glutathione S-transferase (A0A6J1IX51), and probable phospholipid hydroperoxide glutathione peroxidase (A0A6J1K6F2)) (Table S4). DEPs correspond directly to the glutathione metabolism category, emphasizing that glutathione is a key factor in seed deterioration. In plant seeds, glutathione metabolism has a significant protective function as a regulator of ROS levels since the activity of other antioxidant enzymes, such as superoxide dismutase and catalase, is relatively low.^65−67^ To understand the effect of seed deterioration on the glutathione content, total glutathione was investigated using a glutathione assay kit. Evidence of a relationship between glutathione and seed aging has been reported. The influence of seed aging on the oxidation of reduced glutathione (GSH) and the function of GSH oxidation in aging-induced deterioration were investigated in tomato seeds. It was indicated that GSSG formation could only partly explain the decline in GSH, resulting in a loss of total glutathione.^68^ Similarly, our study found that total glutathione levels declined significantly after 15 days of artificial aging. There have been reports on the influence of aging on the level of glutathione. In the investigation of ascorbate-glutathione activity in soybean seed after artificial aging, there was a decrease in total ascorbic acid (ASC) and GSH content, indicating that artificial aging reduced ASC-GSH cycle activity.^23^ According to a study of the activity levels and expression of antioxidant enzymes in the ascorbate glutathione cycle in artificially aged rice seed, GSH plays a role in the removal of H_2_O_2_. It was reported that reducing the ascorbate glutathione cycle in aged rice embryos lowered their ability to scavenge ROS. Total GSH, reduced GSH, and GSSG levels in aged rice seeds were all decreased. These studies indicate that a reduced cycle of the ascorbate glutathione cycle, which promotes cellular ROS accumulation, may lead to seed aging.^69^ Our findings suggested that glutathione metabolism was significantly involved in ridge gourd seed, which could contribute to the aging process. It was suggested that the decrease in total glutathione content was due to a decrease in glutathione-related enzyme activities.

To investigate biological mechanisms during the artificial aging of the ridge gourd seed, protein–protein interactions were performed using proteomic data based on STRING analysis. We found that ubiquitin–proteasome-related DEPs changed throughout seed aging. Proteasome-associated DEPs were found downregulated on 15 days, including proteasome subunit alpha (A0A6J1IYS4, A0A6J1IKP1, A0A6J1IGU1), 26S proteasome non-ATPase regulatory subunit 2 homologue (A0A6J1JN06, A0A6J1HUY5), and ubiquitin receptor RAD23 (A0A6J1IZ65) (Table S3). For 30 days, proteasome-associated DEPs including probable 26S proteasome non-ATPase regulatory subunit 3 (A0A6J1HXW8) were upregulated. In addition, ubiquitin receptor RAD23 (A0A6J1HT74) was shown as a downregulated protein on day 30 (Table S4). Based on STRING analysis, proteasomes were visualized on downregulated DEPs of 30 days, which showed their importance in CC functions (Figure S2). The ubiquitination pathway is complicated, and the entire process is regulated by cellular signaling processes.^70^ The turnover of proteins occurs primarily via the ubiquitin-proteasome system (UPS) and autophagy. Ubiquitin-labeled proteins are targeted for degradation by the UPS and recognized by the proteasome. Following that, identified proteins are unfolded and destroyed by the proteasome in an ATP-dependent system.^71−73^ In this work, the proteomic and bioinformatic study demonstrated that UPS proteins were highly expressed after 30 days of artificial aging. These results indicate that proteins were degraded as a result of the aging process, which was influenced by the ubiquitin–proteasome system.

## Conclusions

In this study, we report a comparative proteomic study of the effect of artificial aging on ridge gourd seeds. Quantitative proteomic analysis was utilized to investigate protein dynamics during seed deterioration. We compared the proteome profiles of nonaging versus artificially aged ridge gourd seed. Proteomic data indicated that the glutathione system was one of the major contributors to the aging process in the ridge gourds. In addition, the total sucrose content was found to decrease during the artificial aging of ridge gourd seeds. Overall, our present findings provided better insight into the regulatory mechanisms related to the seed aging process of ridge gourd in which aconitase, glutathione, proteasome, ribosome, and energy metabolic utilization were prominent. The research presented here has advanced our comprehension of ridge gourd seed proteomics and the mechanism of the aging process. Moreover, this knowledge also had the potential to enhance the development of methods for monitoring seed quality and production.

## Material and Methods

### Plant Materials and Artificial Aging Test

Commercial ridge gourd seeds with 98% genetic purity were purchased from a local distributor. The seeds were stored in aluminum foil-packed bags at room temperature and were produced in 2022. Seeds were treated with high temperature and humidity to perform the artificial aging method. Briefly, seeds were exposed to 40 °C and 90% air humidity for different time periods (0, 6, 10, 15, 21, 25, and 30 days). Seeds without treatment were used as nonage control.^74^ A total of 200 dry seeds were each taken from three independent experiments. For each experiment, seed samples were divided equally into two groups for the germination test and the proteomic study in which the samples were immediately frozen in liquid nitrogen and stored at −80 °C for further proteomic and biochemical analyses.

### Germination Test

The germination test was performed according to the recommendation of the International Seeds Testing Association (ISTA).^75^ Three replicates of 100 seeds for each accelerated aging period were grown on wet filter paper and allowed to germinate in a growth control chamber at 30 °C in a 12/12 h light/dark cycle. After 7 days, seeds with radicle protrusion of at least 1 mm were considered germinated. The seed germination rate was calculated using the formula:
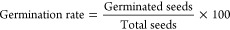


### TZ Test

TZ seed staining is a biochemical test that distinguishes between live and dead seeds based on the activity of respiration enzymes in seeds.^76^ The activity of dehydrogenase enzymes increases, resulting in the release of hydrogen ions, which convert the colorless TZ salt solution (2,3,5-triphenyl tetrazolium chloride) to a chemical molecule known as formazan. The TZ staining patterns were evaluated using 10 seeds of each 0 and 15 days. In preparation for the test, the seeds were soaked in water for 1 h and horizontally cut to remove the seed coat. Seeds were then immediately submerged in the TZ solution and kept in the dark at room temperature for 24 h. The solution was then discarded, and the seeds were rinsed with cool water. Enough water was retained after rinsing to keep the seeds immersed. The stained seeds were stored in a refrigerator until the time of examination.^77^

### Sample Preparation for Proteomic Analysis

The seed proteins were extracted using a combination of chemical and mechanical methods to obtain the highest protein yield. All seeds were added to lysis buffer (1% SDS, 10 mM NaCl, 5 mM dithiothreitol, 50 mM HEPES-KOH, pH 8.0) and incubated at 50 °C for 10 min. After cooling, the sample was subjected to 5 mm stainless steel balls and homogenized for 1 min at 30 Hz using a TissueLyser II (QIAGEN, Germany). The extracted protein solution was obtained by centrifugation at 10,000 × *g* for 10 min, and the supernatant was collected and stored at −20 °C until further processing. The sample preparation and cleanup were conducted following a previously published publication without any modification.^78^ Quality control for sample preparation, digestion, and cleanup was done using 0.2 μg of bovine serum albumin (*n* = 2) for evaluating digestion efficiency.

### LC–MS/MS Method for Proteomic Analysis

The tryptic peptides were analyzed using UltiMate 3000 ultrahigh-pressure system (Thermo Scientific, Germany) coupled to TripleTOF 6600+ (AB SCIEX, Canada). The peptides were separated in reversed-phase (RP) chromatography using Acclaim PepmMap100 C18 (Thermo Scientific, Lithuania) at a column temperature of 55 °C. The dimensions of the columns were 75 μm internal diameter, 15 cm length, and 1.9 μm particle size. Liquid chromatography (LC) conditions: mobile phase A (A) composed of 0.1% formic acid (FA) in water and mobile phase B (B) comprising 80% acetonitrile with 0.1% FA. The samples were loaded onto a column that was first linear gradient separated according to 95 min from 3 to 35% B from the nano-LC system at a constant flow rate of 300 nL/min. The analytical column was regenerated at 90% B for 10 min and re-equilibrated at 5% B for 15 min. Precursor masses were collected in the mass range of 400–1500 *m*/*z* with 250 ms in “high sensitivity” mode. Further fragmentation of each precursor spectrum occurred, with a maximum of 30 precursors per cycle. SWATH-MS data for individual samples were acquired on the LC configuration the same as described above. SWATH-MS acquisition was carried out in data-independent acquisition mode. The MS1 spectra were collected in the mass range of 400–1250 *m*/*z* in “high sensitivity” mode. The variable Q1 isolation windows are optimized based on the spectral library using SWATH Acquisition Variable Window Calculator.^79^ Collision energy was different for each window. Injections of three biological replicates and three technical replicates from each biological replicate were performed.

### Data Processing for Peptide and Protein Identification and Quantification

The raw MS-spectra files (.wiff) were subjected to analysis using the Paragon Algorithm within the ProteinPilot Software.^80^*Cucurbitaceae* protein database sourced from UniProtKB (February 2022) was utilized in the Paragon analysis. Protein identification was performed with a detection threshold of [Unused ProtScore (Conf)] ≥ 0.05, maintaining a stringent 1% false discovery rate (FDR) and requiring a minimum of ≥10 peptides per protein. The protein and peptide comparisons exhibiting >20% coefficient of variation (C.V.) between the replicates were excluded. Subsequently, both the library and SWATH-MS data were imported into the SWATH processing microapp within the PeakView software for subsequent analysis and interpretation. All experiments were carried out in three independent replicates (*n* = 3) with three technical replications for each replicate, and all data were expressed as the means ± standard deviation. The statistical significance was determined by Duncan’s multiple range test (*p*-value < 0.05). For the pairwise comparisons during the proteomic analysis, we performed one-way analysis of variance (one-way ANOVA) at the protein-level analysis with two multiple testing correction methods including the Bonferroni and the Benjamini–Hochberg FDR corrections using the ProteinPilot software.

### Bioinformatic Analysis

GO analysis was employed to classify protein into three terms by Blast2GO Annotation via OmicsBox software version 2.2.4. KEGG is the pathway database^81,82^ which was performed through OmicsBox software KEGG pathway analysis plugin in the functional analysis module. The protein–protein interaction (PPI) was identified using the STRING database online server version 12.0 (https://string-db.org).^83^

### Aconitase Activity Assay

Mitochondrial aconitase is an enzyme within the TCA cycle that facilitates the conversion of citrate to isocitrate.^84^ We investigated seed mitochondrial aconitase activity using the Aconitase Assay Kit (MAK337, Sigma-Aldrich, USA) according to the manufacturer’s instructions. Briefly, 100 mg of each 0 and 15-day seeds were extracted. The extraction for bioactivity assay was described as follows: samples were extracted using a Vibra-Cell VCX750 ultrasonic processor (Sonics, USA) on ice for 5 min (power of amplitude 20%, pulse on 3 s, pulse off 7 s, repeat 30 times). Samples were centrifuged at 20,000 × *g* for 10 min at 4 °C, and the supernatant was collected for assay. The sample was stored at −80 °C until analysis. The reaction mix included the substrate citrate, NADP/MTT solution, which, following the generation of isocitrate, is converted to an intensely purple formazan with an absorption maximum of 565 nm. The absorption was first observed after an incubation time of 10 min (A565)_10_, followed by an incubation time of 30 min (A565)_30_ using a microplate reader (FlexStation 3, Molecular Device, USA). All samples had a protein concentration of 1 μM, and all experiments were measured in triplicates. The aconitase activity of the samples was then determined using the following equation:
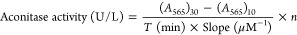


The reaction time is in minutes (*T*_min_), and *n* is the dilution factor. The slope value was determined from the standard curve. Finally, the values for the aconitase activity were normalized to the highest value.

### Determination of Total Glutathione Content

In this study, we employed the CheKine Micro Total Glutathione (T-GSH) Assay Kit (HTB1670, Abbkine, China) to quantitatively assess the total glutathione levels. The test is based on the reaction of reduced *glutathione* (GSH) with 5,5′-Dithiobis-2-Nitrobenzoic Acid (DTNB), which results in the formation of the 2-nitro-5-diol benzoic acid. This material is yellow and absorbs at 412 nm. The developed oxidized *glutathione* (GSSG) can be reduced back to GSH by glutathione reductase, and GSH can then react with DTNB to make an additional 2-nitro-5-thiobenzoic acid. As a result, the recycling mechanism significantly enhances the sensitivity of total glutathione detection. The sample preparation of seed for T-GHS was performed following the manufactured procedure. Briefly, 100 mg of seed was meshed in extraction buffer and sonication using Vibra-Cell VCX750 ultrasonic processor (Sonics, USA) on ice for 5 min (power of amplitude 20%, pulse on 3 s, pulse off 7 s, repeat 30 times). The supernatant was collected for assay by centrifugation at 8000 × *g* for 10 min at 4 °C. Two microliters of samples were mixed with 200 μL reaction mixtures and incubated in darkness for 10 min at 37 °C. Total glutathione content was measured at the absorbance of 412 nm using a spectrophotometer (FlexStation 3, Molecular Device, USA) to determine the slope of the resulting standard curve. The glutathione concentration measured is the sum of reduced and oxidized glutathione in the sample. All results were presented as means standard deviation, and the experiments were performed in at least three independent repetitions (*n* = 3), each with three technical replications.

### Analysis of Sugar Contents

Sugar (glucose, fructose, and sucrose) content of ridge gourd seed was determined using Sucrose/d-Fructose/d-Glucose Assay Kit (K-SUFRG, Megazyme, Ireland) according to the manufacturer’s protocol with some adjustments. Briefly, seed extracts were prepared previously described in the aconitase activity assay. The sugar content analysis was conducted using 96-well plates. First, 2 μL of β-fructosidase reagent was added to the blank and sample well, and then 1 μL of the extracted sample was added to the sample and free sugar sample cuvettes, mixed, and allowed to stand for 5 min at 30 °C. Subsequently, 200 μL of distilled water was added, followed by 1 μL of buffer and 1 μL of NADP/ATP solution, mixed, and incubated for another 3 min. Subsequently, 20 μL of the HK/G6P-DH solution was added to each well, and the absorbance was measured using a spectrophotometer (FlexStation 3, Molecular Device, USA) at 340 nm. Finally, the absorbance was then measured at 340 nm after 0.2 μL of phosphoglucose isomerase (PGI) solution had been added to the well containing the free sugar sample and the blank free sugar. The free sugar concentration was determined to be 5 mg/mL.

### Statistical Analysis

All biochemical test studies were performed with at least three biological replications (*n* = 3) and three technical replications for each biological replicate, with all results presented as means standard deviation. The statistical significance was determined by Student’s *t* test (*p* < 0.05) using Microsoft Excel (Microsoft Corporation, Redmond, WA, USA).

## Supporting Information Available

Total protein identification from spectral library of ridge gourd seed (*Luffa acutangula* (L.) Roxb.); significantly upregulated proteins in ridge gourd seed (*Luffa acutangula* (L.) Roxb.) among days 0, 15, and 30; significantly downregulated proteins in ridge gourd seed (*Luffa acutangula* (L.) Roxb.) among days 0, 15, and 30; and KEGG pathway enrichment analysis of up/downregulated proteins expression in days 15 and 30 (XLSX)
Biological process classifying DEPs according to their functions; molecular function categorizing DEPs according to their functions; gene ontology classifying DEPs according to CC; STRING database revealing the protein–protein interaction of the downregulated DEPs of 15 days; and STRING database revealing the protein–protein interaction of the downregulated DEPs of 30 days (PDF)
