# Supplementary material for: Comparative Proteomic Analysis of Ridge Gourd Seed (Luffa acutangula (L.) Roxb.) during Artificial Aging
Source: ACS Omega. 2024 May 30;9(23):24739–50. doi: 10.1021/acsomega.4c01270 (PMC11171090; doi:10.1021/acsomega.4c01270)
Supplement: Supplementary file 2 — ao4c01270_si_002.pdf [file ao4c01270_si_002.pdf]

Supporting information: figures S1-S5

Comparative proteomic analysis of ridge gourd seed (*Luffa acutangula* (L.) Roxb.) during artificial aging

Jakkaphan Kumsab[1], Yodying Yingchutrakul[1], Nattapon Simanon[1], Chonchawan Jankam[1], Chutima Sonthirod[1], Sithichoke Tangphatsornruang[1], Chutikarn Butkinaree[1,*]

1 National Center for Genetic Engineering and Biotechnology, National Science and Technology Development Agency, Pathum Thani, Thailand

* Corresponding author

E-mail: chutikarn.but@nstda.or.th

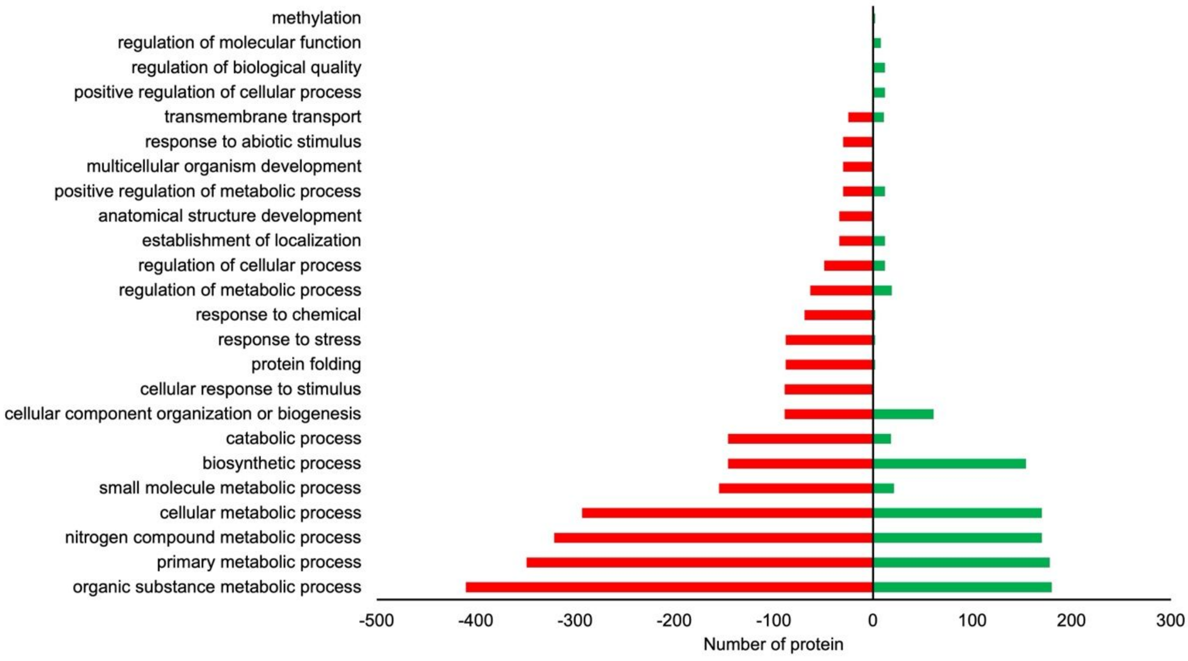

Figure S1 Biological process classifies DEPs according to their functions. The red, and green colors demonstrate the number of downregulated and upregulated proteins.

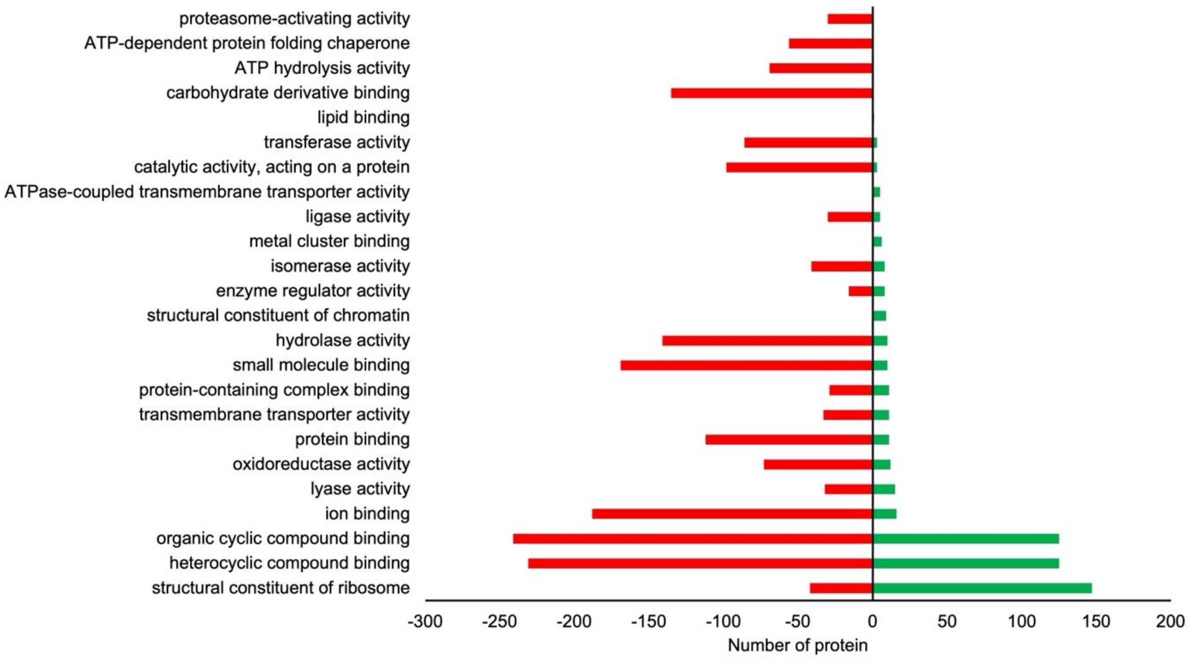

Figure S2 Molecular function categorizes DEPs according to their functions. The red, and green colors demonstrate the number of downregulated and upregulated proteins.

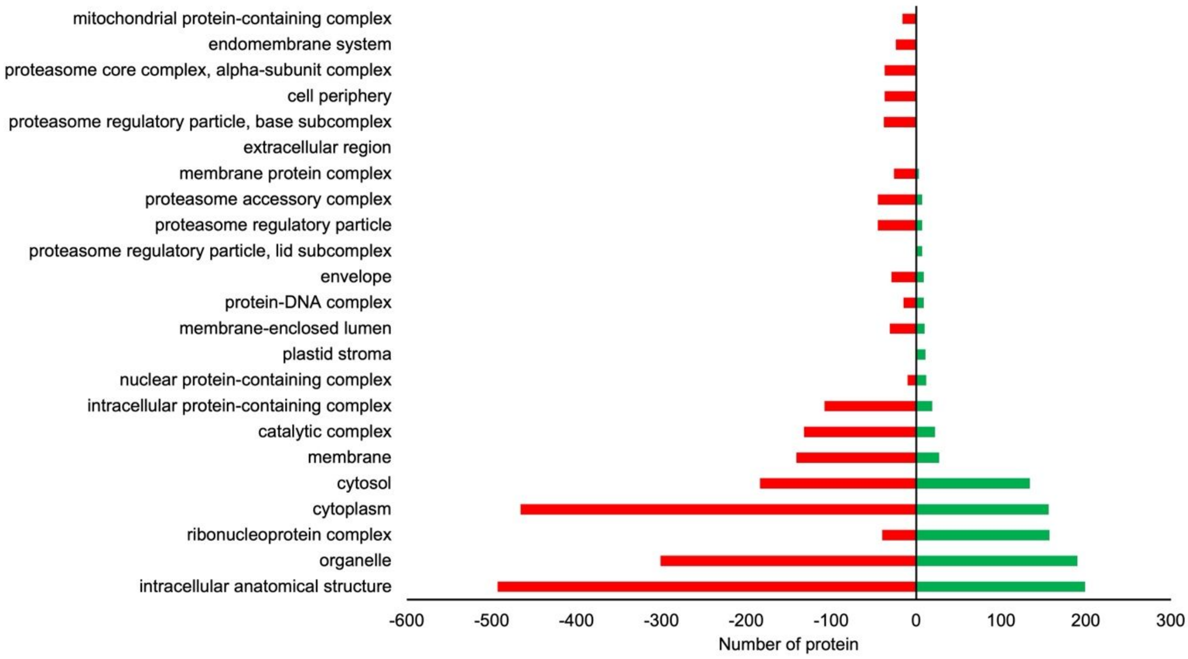

Figure S3 Gene ontology classifies DEPs according to cellular component. The red, and green colors demonstrate the number of downregulated and upregulated proteins.

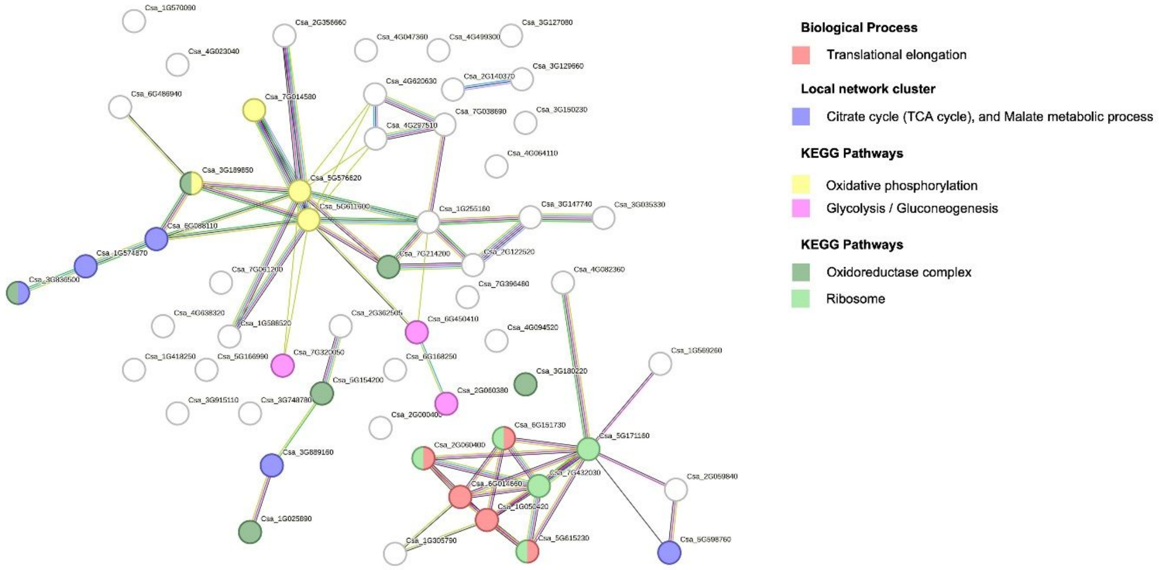

Figure S4 STRING database reveals the protein-protein interaction of the downregulated DEPs of 15d.

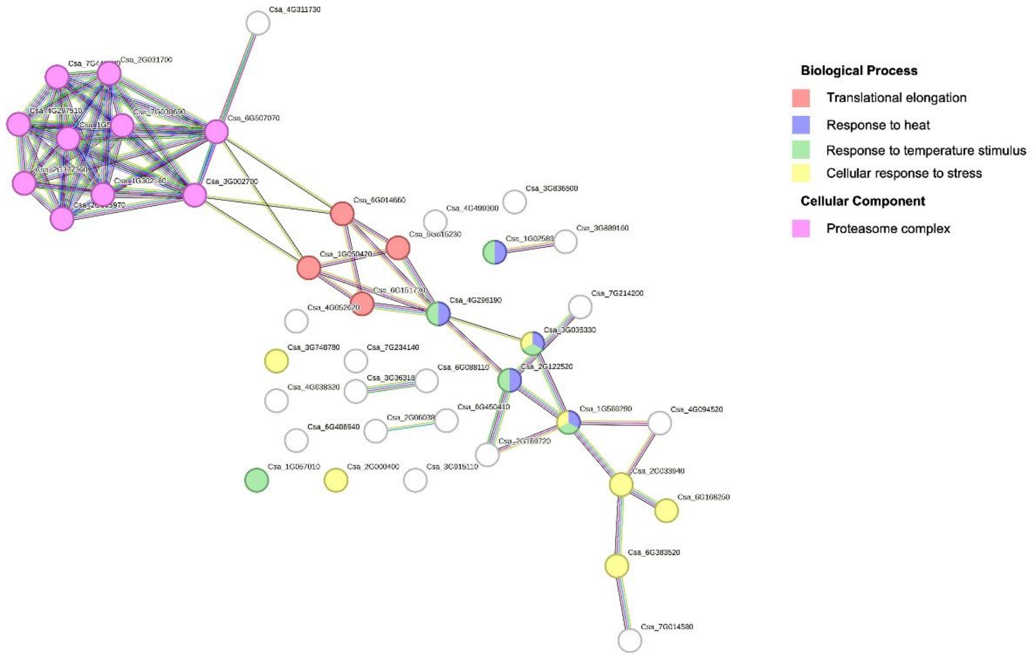

Figure S5 STRING database reveals the protein-protein interaction of the downregulated DEPs of 30d.
